# Glial–Neuronal Interactions in Neurological Disorders: Molecular Mechanisms and Potential Points for Intervention

**DOI:** 10.3390/ijms24076274

**Published:** 2023-03-27

**Authors:** Agata Adamczyk

**Affiliations:** Department of Cellular Signaling, Mossakowski Medical Research Institute, Polish Academy of Sciences, Pawińskiego 5, 02-106 Warsaw, Poland; aadamczyk@imdik.pan.pl; Tel.: +48-22-608-6572

Neurons have long been central to the study of cellular networks in the nervous system. However, integrative approaches developed over the last two decades, at the genetic, molecular, cellular, and synaptic levels, have resulted in a fundamental paradigm shift in our understanding of the structure and function of the central nervous system (CNS). Today, there is no doubt that a functional brain depends not only on neurons, but also on non-neuronal glial cells, such as astrocytes, microglia, and myelin-forming oligodendrocytes. These three kinds of glial cell associate closely with neurons and modulate their properties in many ways [1,2,3].

Glial cells are primarily involved in the arrangement of neuroinflammation processes, in both deleterious and beneficial ways; they also participate in synapse formation, maturation, and elimination, contributing to the development of neural circuits [4], which is essential to maintaining proper neural processing and function in the CNS. The existence of close morphological and functional associations between astrocytes or microglia and synapses gave rise to the ‘tripartite’ and ‘quad-partite’ synapse models (Figure 1); these include perisynaptic glial processes, which are integral to the synapse, in addition to classic ‘bipartite’ pre- and postsynaptic neurons [5,6]. Microglia release cytokines and soluble factors, such as brain-derived neurotrophic factor (BDNF), glutamate, TNFα, IL1β, glycine and L-serine; these can affect basal neurotransmission and synaptic plasticity via direct action against neurons, or can act on astrocytes, leading to increased glutamate release. In addition to glutamate, other gliotransmitters, such as gamma-amino butyric acid (GABA), ATP, and D-serine, bind to their respective receptors on neurons to modulate synaptic transmission and plasticity [7]. 

Any imbalance in the release of these soluble factors or gliotransmitters could affect synaptic structure and function. Therefore, two-way communication between neurons and glial cells, as well as between glia, plays a critical role in brain functionality. Recent studies have revealed the prominent and often causative roles of aberrant neuronal–glial interactions in the development and progression of most known CNS pathologic conditions, including neurodegenerative diseases such as Alzheimer’s disease (AD), Parkinson’s disease (PD), amyotrophic lateral sclerosis (ALS), spinal muscular atrophy (SMA), multiple sclerosis (MS), and various neurodevelopmental disorders such as autism spectrum disorders (ASD) and depression [8]. However, our understanding of the extent of complex glial–neuronal interactions remains limited.

Therefore, the major goal of this Special Issue is to discuss the key functional properties of intercellular signaling in neuronal–glial networks to understand brain function under both physiological and pathological conditions. Novel therapeutic strategies based on the modulation of the immune system are also proposed.

Several articles focus on the functional relationship between neurons and glia in neurodegenerative disorders, such as AD, PD, ALS, and prion diseases. Gul-Hinc et al. [9] investigated whether and how astroglial cells alleviate Zn neurotoxicity in cultured neuronal cholinergic SN56 cells in thiamine-deficient media; their aim was to understand astrocyte–neuron interactions in AD pathology that is compounded by thiamine deficiency, especially among elderly people who are malnourished. Multiple similarities exist between conventional thiamine deficiency and AD, in that both are associated with cognitive deficits and alterations in brain glucose metabolism. Reduced thiamine levels can drive AD-like abnormalities, such as amyloid plaque formation and tau protein hyperphosphorylation, as well as memory deficits [10]. These in vitro studies provide evidence that astroglial cells are capable of protecting neuronal cells against Zn toxicity in thiamine deficiency, thus shedding new light on the role of astroglia in the pathology of AD associated with thiamine deficiency. Research by Czapski et al. [11] confirmed the significance of neuronal–immune system cross-talk in neurodegeneration. The authors provide insight into the global gene expression pattern in the hippocampus after acute and mild systemic inflammatory responses. The results demonstrated that some genes that are activated in the brain during systemic inflammation are also upregulated in the brains of AD patients, suggesting that early changes in the expression of immune-response genes may play a role in AD pathogenesis. In Ruan et al. [12], a correlation between microglial activation and dopaminergic neuron death was found in a PD mouse model. The authors suggested that the activation of microglial metalloproteinase-2 and -9 (MMP-2/-9) mediated blood–brain barrier (BBB) dysfunction, contributing to dopaminergic neurodegeneration. These results extend our understanding of the glial–neuronal relationship in neurodegenerative disorders and provide a novel view of the molecular mechanisms of PD pathology. Studies by Guijarro et al. [13], who examined the role of glial cells in a natural neurodegenerative model of scrapie, provided evidence that the modulation of neuroinflammation by anti-inflammatory drugs might be a novel therapeutic approach to addressing prion diseases and other neurodegenerative disorders classified as prion-like diseases. Despite fundamental differences in their disease course and outcomes, neurodegenerative and neuropsychiatric disorders, such as ASD and depression, present common traits in their molecular pathomechanisms that result from altered glial–neuronal interaction. Cieślik et al. [14] and Gąssowska-Dobrowolska et al. [15] emphasized that environmental stressors, such as maternal infection during pregnancy or prenatal exposure to drugs or toxicants, can induce microglial activation and the pro-inflammatory environment in the brains of offspring, which may cause synaptic alterations and the development of autistic symptoms. Similarly, depression might result from aberrant bidirectional neuronal–glial communication [16], indicating that dysfunctional neuronal–glial interaction may underlie many different neuropsychiatric disorders, including AD, ASD, and depression. Tao and co-workers [17] proposed that lipopolysaccharide (LPS) administration alters the levels of the two metabolites of the kynurenine (KYN) pathway, 3-hydroxykynurenine (3-HK), and kynurenic acid (KYNA), by regulating the homeostasis of microglia and astrocytes, ultimately affecting the neuroplasticity of the central nervous system and leading to depressive-like behavior in mice.

Further, using postnatal dissociated cortical neurons derived from neonatal opossums (*Monodelphis domestica*), the authors of [18] focused on the role of activating transcription factor 3 (ATF3) in the regeneration of CNS neurons after injury. ATF3 is a member of the ATF/cyclic adenosine monophosphate(cAMP)-response element binding protein (CREB) family of transcription factors that modulates the inflammatory response. Petrović et al. showed that ATF3, and other members of ATF/CREB family of transcription factors, play an important role in neuronal differentiation, survival, network formation, and regeneration in response to injury.

Lastly, this Special Issue presents comprehensive reviews that describe glial–neuronal cross-talk in physiology and different brain disorders. Lukiw and Pogue [19] discussed the role of exosomes (EXs) and extracellular microvesicles (EMVs) in communication between glia and neuronal cells. They emphasized the role of EXs and EMVs in the trafficking of selective pathogenic microRNAs (miRNAs), such as miRNA-34a, miRNA-125b, miRNA-146a, and miRNA-155 from astrocytes, and activated pro-inflammatory microglia to target neurons in neurodegenerative diseases such as AD. Jankowska-Kulawy et al. [20], focusing on the role of glial–neuronal cell cross-talk in the regulation of energy metabolism, discussed the molecular pathways involved in the regulation of the main producer of energy, acetyl-coenzyme A (acetyl-CoA), in healthy and diseased brains. They concluded that the identification of metabolic pools of acetyl-CoA in different neuronal and glial compartments is of great significance in target-oriented approaches to the management of neurodegenerative diseases. Two papers, by Lukacova et al. [21] and Maximova et al. [22], focus on the role of glial–neuronal interactions in the pathogenesis and treatment of spinal cord injury and amyotrophic lateral sclerosis (ALS), respectively. A more specific review by Gromadzka et al. [23] focusses on copper dyshomeostasis in Wilson’s disease and other neurodegenerative disorders, such as AD and PD. Alterations in neuronal–glial signaling is suggested to play an important role in the initiation and pathology of migraine. A review by Kowalska et al. [24] focusses on these interactions, especially when seen in familial hemiplegic migraine (FHM) type 1, a variant of the disease caused by a mutation in *CACNA1A*, which encodes the α1A subunit of the P/Q-type voltage-gated calcium channel. Fernández-Blanco et al. [25] concentrated on the unexplored role that astrocytes may play in the impairment of fundamental mechanisms required for memory function, including synaptic and neuronal communication, and focused primarily on the neurodevelopmental disorders associated with intellectual disability, such as Fragile X Syndrome (FXS) and Down Syndrome (DS). Fernández-Blanco and co-workers proposed specific new models of astrocyte alterations, which would explain the perturbations in spine maturation that result in more abundant immature spines in both FXS and DS. The authors stated that astrocytes could become a promising target of new treatments for a number of brain disorders associated with intellectual disability.

This Special Issue aims to change the “neurocentric” view of the mechanisms involved in the onset and progression of several neurological disorders. The articles published herein highlight the key and complex nature of the relationship between glia and neurons, and the role of glial cells in the occurrence of synaptic changes during development, adulthood, and aging. Understanding how these shared pathomechanistic elements operate under different conditions may help identify common targets and therapeutic approaches, which might benefit the development of therapies for neurodevelopmental, neuropsychiatric, and neurodegenerative diseases.

## Figures and Tables

**Figure 1:**
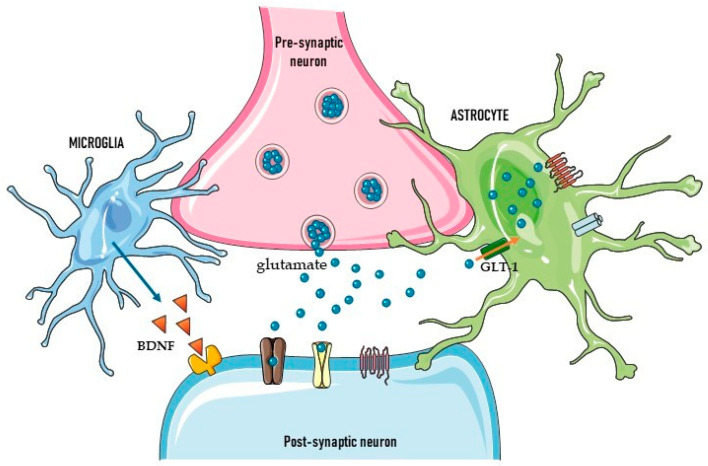
Scheme of a “quad-partite” glutamatergic synapse. The processing of information in synapses is not only defined by neurons, but also by astrocytes which enwrap and microglia that dynamically interact with synapses. Astrocytes respond to glutamate and other neurotransmitters such as GABA, noradrenaline, acetylcholine or ATP. Then astrocytic calcium waves can feedback to influence neuronal responses and control synaptic strength through the release of mediators such as glutamate, ATP, D-serine etc. Thus, astrocytes maintain glutamate homeostasis and the basal excitability of neurons. Microglia release soluble factors such as BDNF, TNFα, glycine, L-serine etc., which affect basal neurotransmission and synaptic plasticity (i.e., LTP) via direct action on neurons or indirectly via astrocytes.
